# Anxiety is associated with cognitive impairment in newly-diagnosed Parkinson's disease

**DOI:** 10.1016/j.parkreldis.2017.01.001

**Published:** 2017-03

**Authors:** Nadeeka N.W. Dissanayaka, Rachael A. Lawson, Alison J. Yarnall, Gordon W. Duncan, David P. Breen, Tien K. Khoo, Roger A. Barker, David J. Burn, ICICLE-PD study group

**Affiliations:** a The University of Queensland, UQ Centre for Clinical Research, Brisbane, Australia; b Department of Neurology, Royal Brisbane & Women's Hospital, Brisbane, Australia; c School of Psychology, The University of Queensland, Brisbane, Australia; d Institute of Neuroscience, Newcastle University, Newcastle Upon Tyne, UK; e Centre for Clinical Brain Sciences, University of Edinburgh, Edinburgh, UK; f John van Geest Centre for Brain Repair, University of Cambridge, Cambridge, UK; g School of Medicine & Menzies Health Institute Queensland, Griffith University, Australia

**Keywords:** Anxiety, Mild cognitive impairment, Parkinson's disease, Cognitive subtypes

## Abstract

**Introduction:**

Anxiety and mild cognitive impairment (MCI) are prevalent non-motor manifestations of Parkinson's disease (PD). While few studies have demonstrated a possible link between cognitive dysfunction and anxiety in PD, to our knowledge, no studies have directly examined the association between them. This study investigated the association between anxiety and cognitive deficits in newly diagnosed PD patients.

**Methods:**

Patients with newly diagnosed PD (N = 185) were recruited from community and outpatient clinics. Anxiety was assessed using the Unified Parkinson's Disease Rating Scale (MDS-UPDRS) clinician rated anxiety item, which has previously been validated against a standardized criteria for the diagnosis of anxiety disorders in PD. Participants scoring ≥2 were classified as anxious. A threshold of 1 SD below normative values (obtained from controls) was used to define cognitive impairment. Impairments in specific cognitive domains were identified as being >1 SD below controls in ≥1 test per domain.

**Results:**

After controlling for age, education and motor severity, patients with anxiety were three times more likely to have cognitive impairment compared to those without anxiety (OR = 3.0, 95% CI = 1.2–7.3, p < 0.05). Patients with anxiety were more than twice as likely to be classified as having cognitive impairment due to impairment in the memory domain compared with PD without anxiety (OR = 2.3, 95% CI = 1.0–5.1, p < 0.05), whilst no associations were found between anxiety and performance on other cognitive domains.

**Conclusion:**

This study shows an association between anxiety and cognitive impairment (specifically memory impairment). Examining the neural basis of this association warrants future research in this developing field.

## Highlights

• Anxiety was associated with mild cognitive impairment (MCI) in PD.
• Patients with anxiety were three times more likely to have cognitive impairment.
• Anxiety was specifically associated with impairments in the memory domain.

## 1 Introduction

Anxiety and mild cognitive impairment (MCI) are common in Parkinson's disease (PD) [1]. A recent systematic review estimated that the prevalence of anxiety in PD to be 31%, based on the Diagnostic and Statistical Manual of Psychiatric Disorders (DSM) criteria or scoring above thresholds on validated rating scales [2]. Cognitive deficits are also commonly observed at the time of PD diagnosis, with many patients ultimately developing dementia [3]. Mild cognitive impairment in PD (PD-MCI) may represent a transitional state between a cognitively intact stage and dementia (PDD) [4].

Studies examining the association between anxiety and PD-MCI are limited. To our knowledge, there are no studies that have directly examined the relationship between these two primary neuropsychological complaints in newly diagnosed PD patients. Such an approach would be useful in the clinical management of these non-motor symptoms and the identification of neuropsychological subtypes in PD. It has been suggested that cognitively impaired PD patients are more likely to use anti-anxiety medication than those without [5] and that pharmacological treatment of PD-MCI using rivastigmine has been shown to reduce concomitant anxiety [6].

A few studies have demonstrated a possible link between cognitive dysfunction and anxiety in PD [7], [8], [9], [10], [11], although none directly investigated this association nor examined the interdependence between anxiety and cognitive subtypes in PD. This study investigated the role of anxiety in cognitive impairment, and specifically, the cognitive domains affected in this latter state in newly diagnosed PD. It was hypothesised that anxiety is associated with cognitive impairment based on studies in older adults [12], [13] with impairments in memory and executive functioning being those domains most positively associated with anxiety.

## 2 Methods

### 2.1 Participants

Newly diagnosed PD patients were recruited as part of the Incidence of Cognitive Impairment in Cohorts with Longitudinal Evaluation in Parkinson's Disease (ICICLE-PD) study [14]. PD patients were recruited from community and outpatient clinics in Newcastle-upon-Tyne, Gateshead and Cambridgeshire, UK. Inclusion criteria involved having a diagnosis of idiopathic PD according to the Queen's Square Brain Bank Criteria [15] between 1st June 2009 and 31st December 2011. Exclusion criteria comprised: significant cognitive impairment as defined by scoring <24 in the Mini Mental State (MMSE) or a pre-existing diagnosis of dementia, insufficient command of the English language to complete assessments, dementia with Lewy bodies (DLB), parkinsonism due to another cause, drug-induced parkinsonism, progressive supranuclear palsy, multiple system atrophy or corticobasal degeneration.

Normative data for neuropsychological assessments was provided from age-matched healthy control subjects (n = 99). Carers and spouses were not recruited as control subjects in order to minimize potential bias. Controls were recruited from the general population in the North East of England through local advertising and community to ensure controls were unrelated to PD participants.

This study was approved by the NHS Local Research Ethics Committee, Newcastle and North Tyneside 1. All participants gave written informed consent prior to study inclusion.

### 2.2 Assessments

Demographic information, including age, sex and education, was collected. All subjects underwent a medical assessment, which included an interview with a movement disorders specialist. Details of disease duration from diagnosis, level of education, co-morbidities, and medication use were recorded. All patients with PD on antiparkinsonian medications were evaluated in the “on” motor state; this was determined by the researcher and from asking participants about their response to antiparkinsonian medication. Motor disability and disease severity were rated with the MDS revised Unified Parkinson's Disease Rating Scale (MDS-UPDRS) part III and Hoehn and Yahr scale, respectively [16], [17]. Levodopa equivalent dose (LED) was calculated for all dopaminergic medications using the conversion factors outlined by Tomlinson et al. [18].

Participants completed the National Adult Reading Test (NART) [19] as a measure of pre-morbid IQ, and the Geriatric Depression Scale (GDS-15) [20]. Anxiety was assessed using the anxiety item from the MDS-UPDRS [17]. This clinician-rated anxiety assessment has been recently validated against DSM-IV criteria for anxiety disorders with high validity (area under the Receiver Operating Characteristic curve was 0.94 and suggested an optimal cut-off score of ≥2) (unpublished data. Doctor of Psychology thesis completed by Elizabeth Torbey and supervised by Dissanayaka. Manuscript is currently in preparation). Participants were classified as anxious if they scored ≥2.

### 2.3 Neuropsychological testing

Participants completed a schedule of neuropsychological tests across five cognitive domains. Global cognitive function was assessed with the MMSE [21] and Montreal Cognitive Assessment (MoCA) [22]. Attention was measured using Power of Attention (PoA) and Digit Vigilance Accuracy from the Cognitive Drug Research (CDR) battery [23]. PoA is a composite score of Simple Reaction Time, Choice Reaction Time and Digit Vigilance reaction; a higher score indicating greater impairment. Memory was assessed using Pattern Recognition Memory (PRM), Spatial Recognition Memory (SRM) and Paired Associates Learning (PAL) from the computerised Cambridge Neuropsychological Test Automated Battery (CANTAB) [24]. Executive function was determined using the One Touch Stockings (OTS) task from the CANTAB [24], phonemic fluency (words beginning with F in one minute) [25] and semantic fluency (naming animals in 90 s) [26]. As a measure of visuospatial function the pentagon copying item of the MMSE was graded using a modified 0–2 rating scale [27]. Language was assessed using the naming (0–3) and sentence (0–2) subsets from the MoCA.

Participants were classified as having PD-MCI using modified Level II MDS criteria [28], as our schedule of neuropsychological tests preceded these criteria, as described previously [14]. PD-MCI participants were impaired in at least two neuropsychological tests, either on two tests within one cognitive domain, or at least one test in more than one domain. Subjective cognitive and functional independence of the participants was determined through semi-structured interviews with participants and/or their carers. MDS criteria specifies impairment should be between 1 and 2 SD below normative values; as this was an exploratory analysis in newly-diagnosed patients, 1SD, 1.5SD and 2SD below normative values (controls) were each used as cut-offs for PD-MCI. Participants with PD-MCI were further classified by impairments in specific cognitive domain, determined by > 1SD, >1.5SD or >2SD, as appropriate, below controls in ≥1 test per domain, in keeping with PD-MCI criteria [28].

### 2.4 Analysis

Statistical analyses were performed using SPSS (Version 21.0; SPSS, Inc., Chicago, IL). Data were examined for normality of distribution with visual histograms and the Kolmogorov-Smirnov test. Comparison of means between the two groups was performed using independent t-tests or Mann-Whitney U-tests, as appropriate. For comparisons of more than two groups, one-way ANOVA or Kruskal-Wallis tests were used as appropriate. Chi-squared tests were used to assess binary outcome variables. The significance level for all statistical tests was set at α = 0.05 (two-tailed). As this was an exploratory study, corrections for multiple comparisons were not made.

Hierarchical regression was used to determine significant predictors of PD-MCI. Backwards stepwise logistic regression was used to produce a basic model of predictors involving: age, sex, years of education, LED, GDS-15 and MDS-UPDRS III. Non-continuous data were dichotomised using the median. Non-significant predictors were excluded. Significant predictors were then included and anxiety was added to the model.

## 3 Results

Two hundred and nineteen participants completed assessments; subsequently seven participants were re-diagnosed as not having idiopathic PD and were excluded from any further analyses. A further 27 participants did not complete the anxiety item of the MDS-UPDRS as it was introduced after the study had commenced and were excluded from the analysis, leaving 185 PD participants. Participants had a mean disease duration of 5.6 ± 5.2 months; 17% (n = 32) of participants were drug naïve (Table 1). 21% (n = 41) of participants were prescribed anxiolytics and/or antidepressants, comprising: 8% (n = 15) tricyclics, 6% (n = 11) selective serotonin reuptake inhibitors (SSRIs), 3% (n = 6) benzodiazepines, 2% (n = 3) serotonin-norepinephrine reuptake inhibitors (SNRIs), 1% (n = 2) mirtazapine; and 3% (n = 3) were prescribed more than one medication from these groups.

Participants with anxiety (25%) were compared to participants without anxiety (Table 1). Compared to patients without anxiety, anxious PD patients were significantly younger (62.2 ± 8.7 vs. 67.2 ± 3.2, p < 0.01), had higher LED (231.1 ± 192.6 vs. 165.2 ± 145.1, p < 0.05) and GDS-15 scores (4.1 ± 3.2 vs. 2.5 ± 2.2, p < 0.01). There were no significant differences in disease duration, motor severity, years of education, NART score, global cognition or use of anxiolytics and/or antidepressants between anxious and non-anxious PD patients (p > 0.05, for all). There were no significant differences in the neuropsychological scores of participants with anxiety and on treatment with anxiolytics and/or antidepressants compared to those not in receipt of such medications (Supplementary Table 1). Chi-squared tests showed that there were significantly more anxious participants with cognitive impairment using the 1SD cut-off than non-anxious participants (78.7% vs. 61.6%, p < 0.05). However, there were no significant differences using the 1.5SD or 2SD cut-off (p > 0.05, Table 1).

### 3.1 Predicting cognitive impairment

Univariate predictors of PD-MCI were determined using logistic regression. Age, fewer number of years in education and increased motor severity were significant predictors of cognitive impairment at 1SD (p < 0.001 for all, Table 2). Univariate analysis showed that newly diagnosed PD participants with anxiety were more than twice as likely to have cognitive impairment at 1SD (OR = 2.3, 95% CI = 1.1–5.0, p < 0.05). Hierarchical regression was used to produce a basic model. Consistent with the univariate analysis, age, education and motor severity were significant predictors of cognitive impairment after non-significant predictors were removed. Adding anxiety status to the model revealed that it was a significant predictor of cognitive impairment at 1SD (Table 2). PD patients with anxiety were three times more likely to have cognitive impairment at 1SD compared to PD patients without anxiety, even after controlling for age, education and motor severity (OR = 3.0, 95% CI = 1.2–7.3, p < 0.05). However, anxiety was not a significant predictor when using the 1.5SD or 2SD cut off for PD-MCI (Supplementary Table 2).

To determine whether anxiety was associated with particular domains of cognitive impairment, hierarchical logistic regression was used. Backwards regression was used to determine a basic model for each of the five cognitive domains and non-significant predictors were excluded; anxiety status was then added by forced entry (Table 3). PD patients with anxiety were more than twice as likely to be classified as having cognitive impairment at 1 SD with impaired memory (OR = 2.3, 95% CI = 1.0–5.1, p < 0.05). However, there was no significant association with anxiety and the other cognitive domains (p > 0.05). This analysis was repeated with cognitive impairment as defined using 1.5SD and 2SD cut-offs; and no significant association with anxiety was found (Supplementary Table 3).

## 4 Discussion

To the best of our knowledge, the present study is the first to directly focus on a relationship between anxiety, cognitive impairment and cognitive subtypes in PD in a large cohort of newly diagnosed patients. In our incident PD sample, patients with anxiety were three times more likely to present with cognitive impairment at 1SD than patients without anxiety. However, this result did not persist when more stringent cut-offs were applied.

These results are in line with previous studies that have focussed on cognition in PD and evaluated anxiety as a secondary measure. Using the brief Neuropsychological Inventory to assess anxiety, Monastero et al. [11] suggested a positive association between anxiety and PD-MCI in a sample of 410 non-demented PD patients. In a recent smaller study of 40 patients, it was demonstrated that anxiety, as assessed using the Hospital Anxiety and Depression Scale, was associated with subjective cognitive complaints in PD [10]. A further study showed an association between cognitive clusters and avoidance anxiety using the new and validated Parkinson's Anxiety Scale (PAS), which was not available during the data collection phase of our study [7]. The study aimed to identify cognitive clusters to demonstrate the severity of cognitive impairment in PD, but the authors did not relate anxiety to a specific cognitive cluster or domain.

Another novel aspect of the present study is the investigation of the relationship between anxiety and cognitive domains in PD. We found that anxiety was associated with memory impairment using the 1SD cut-off for cognitive impairment, but not with other cut-offs. An association between anxiety and memory problems has been previously reported in community dwelling older adults without PD [12], [13]. The study by Yochim et al. [13] suggested an association between anxiety and semantic memory using the California Verbal Learning Test in 120 older adults. Beaudreau and O'Hara [12] demonstrated a relationship between anxiety and depression, and both episodic memory and semantic memory as assessed using the Rey Auditory Verbal Learning test and Boston Naming Test second edition, respectively. Memory components assessed in the present study were Pattern Recognition Memory, Spatial Recognition Memory and Paired Associates Learning using CANTAB. Although depression often coexists with anxiety [29], our results did not demonstrate an association between depression and cognitive impairment in general or in relation to a specific subtype. Our findings suggest that the presence of anxiety may have prognostic significance in determining which individuals are more likely to develop PDD, and examining the underlying neural basis for this association warrants future study.

Contrary to previous studies [12], [13], the present study did not demonstrate a relationship between anxiety and executive dysfunction in PD. Yochim et al. [13] suggested such an association, and similarly, Beaudreau and O'Hara [12] demonstrated that anxiety is related to poor performance on a number of executive tasks. Teasing this apart is difficult, as anxiety can affect performance on cognitive tasks without their being an actual cognitive deficit. Future investigations are required to identify the relationship between anxiety in PD and other executive functions.

Limitations of the present study should be noted. Firstly, although validated, our definition of anxiety using the clinician rated MDS-UPDRS anxiety item was brief. We did not make a diagnosis of specific anxiety disorders based on criteria such as the DSM-5 or the PAS as our study design preceded the development of the PAS. Future studies investigating the relationship between PD-MCI and anxiety subtypes should consider using these measures. Secondly, we used modified MDS criteria for PD-MCI as our study predated guidelines, with an unequal number of neuropsychological tests per domain; this included more than two tests for memory and executive function and only one test for visuospatial function. This has implications for allowing us to accurately subtype patients' cognitive impairment, and may also increase the frequency of impairments in some domains. Finally, we did not observe significant findings when using 1.5 or 2SD for PD-MCI. It is likely that the smaller number of participants identified at being impaired at this level reduced the statistical power to detect such an association. Thus the data reported here should be viewed as being exploratory and requires confirmation in a larger cohort.

Whilst increased awareness of cognitive deficits is likely to exacerbate anxiety in PD patients [29], a recent prospective study demonstrated that anxiety and MCI cluster together [8]. The authors showed that a rapidly progressive subtype of PD had a poorer prognosis in terms of cognitive decline and non-motor symptoms, including anxiety, at 4.5 years follow-up. Cognitive dysfunction is irreversible in many cases, however, anxiety is treatable [30] and thus treating it could potentially reduce or delay the onset of cognitive impairment in PD. Therefore, understanding the specific cognitive domains associated with anxiety may assist in the development of tailored therapies for anxious PD patients afflicted by coexisting cognitive deficits. Longitudinal and neuroimaging studies are also required to advance our understanding of the contribution of anxiety to the onset of cognitive deficits, progression of PD and the neurobiological basis of these relationships. Such approaches may assist in the identification of early markers for prodromal dementia in PD.

In conclusion, the present study demonstrated a positive association between anxiety and cognitive impairment in PD, in particular memory deficits. A more comprehensive examination of the association between anxiety subtypes and cognitive domains now merits further research.

## Author roles

NNW Dissanayaka was involved with the conception, organisation, design, review of data analysis, writing of the first draft, and revision.

RA Lawson was involved with coordination of the study, data collection, statistical analysis, interpretation of data and co-drafted the manuscript.

AJ Yarnall, GW Duncan and DP Breen were also involved with coordination of the study, participant recruitment, clinical assessment, data collection and manuscript revision.

TK Khoo was involved with the study design and coordination of the study. He was also involved with participant recruitment, clinical assessment, data collection and manuscript revision.

RA Barker is a principal investigator and co-applicant for the main funding grant. He was involved with the study design and reviewed the manuscript.

DJ Burn is the chief investigator and main applicant for the funding grant. He was involved with the study design, supervised the study, data interpretation and reviewed the manuscript.

## Financial disclosure for the preceding 12 months

Nadeeka N W Dissanayaka is employed by the University of Queensland, Australia. She has received grants from Royal Brisbane & Women's Hospital and Royal Brisbane & Women's Hospital Foundation and Lions Medical Research Foundation.

Rachael A Lawson has previously been supported by grants from the Lockhart Parkinson's Disease Research Fund.

Alison J Yarnall is funded by the Biomedical Research Unit, Newcastle University, and has previously been supported by grants from the Lockhart Parkinson's Disease Research Fund and the Michael J. Fox Foundation (MJFF). She has received honoraria from Teva-Lundbeck and sponsorship from Teva-Lundbeck, UCB, GlaxoSmithKline (GSK), Genus, Britannia Pharmaceuticals Ltd. and AbbVie for attending conferences.

Gordon W Duncan has no financial disclosures.

David Breen has received speaker fees from UCB and Britannia Pharmaceuticals Ltd.

Tien K Khoo has no financial disclosures.

Roger A Barker receives editorial monies from Springer and royalties from Wiley. He has grant support from NIHR; Parkinson's UK; Cure Parkinson's Trust; Rosetrees Trust; MRC; ACT and EU.

David J Burn has received grants from NIHR, Wellcome Trust, and Parkinson's UK. He has received speaker fees from Acadia Pharmaceuticals.

## Financial disclosure/conflict of interest related to this manuscript

ICICLE-PD was funded by Parkinson's UK (J-0802) and Lockhart Parkinson's Disease Research Fund. The research was supported by the National Institute for Health Research (NIHR) Newcastle Biomedical Research Unit based at Newcastle upon Tyne Hospitals NHS Foundation Trust and Newcastle University, and a NIHR Biomedical Research Centre award to the University of Cambridge/Addenbrooke's Hospital. Dr Nadeeka Dissanayaka's fellowship is supported by the Lions Medical Research Foundation competitive research fellowship scheme.

The authors have no other financial disclosures or conflicts of interest relating directly to this work.

## Figures and Tables

**Table 1:** Demographic and clinical characteristics of participants.

	Total cohort n = 185		Anxious n = 47		Non-anxious n = 138		Anxious vs. Non-anxious	
	Mean	SD	Mean	SD	Mean	SD	t/Z	p
*Age, years*	65.9	9.5	62.2	8.7	67.2	9.4	3.2^a^	**0.002**
*Education, years*	12.9	3.6	12.9	3.5	12.9	3.7	0.0^b^	0.963
*NART*	114.1	10.5	112.7	9.8	114.6	10.8	−1.6^b^	0.109
*PD duration (months)*	5.6	5.2	5.6	5.3	5.6	5.1	−0.8^b^	0.937
*UPDRS III*	27.9	12.1	30.8	12.9	27.0	11.7	−1.7^b^	0.095
*Hoehn and Yahr*	1.9	0.6	1.8	0.6	1.9	0.6	−0.7^b^	0.507
*LED (mg/d)*	182.0	160.5	231.1	192.6	165.2	145.1	−2.1^b^	**0.033**
*GDS-15*	2.9	2.7	4.1	3.2	2.5	2.4	−3.2^b^	**0.002**
*MoCA*	25.4	3.4	25.8	2.6	25.3	3.7	−0.2^b^	0.862
*MMSE*	28.7	1.3	28.7	1.3	28.7	1.3	−0.2^b^	0.868
	n	%	n	%	n	%	χ^2^	p
*Sex (male)*	117	63.2	27	57.4	90	65.2	0.9	0.340
*Cognitive impairment 1SD*	122	65.9	37	78.7	85	61.6	4.6	**0.032**
*PD-MCI 1.5SD*	79	42.7	20	42.6	59	42.8	0.0	0.981
*PD-MCI 2SD*	39	21.1	9	19.1	30	21.7	0.1	0.707
*Anxiolytics and/or antidepressant use*	41	22.1	15	31.2	26	18.8	3.5	0.062
*PD drug naive*	32	17.3	4	8.5	28	20.3	3.4	0.065

SD = Standard deviation, NART = National Adult Reading Test, UPDRS III = Movement Disorders Society-Unified Parkinson's Disease Rating Scale Part III, LED = Levodopa equivalent dose, GDS-15 = Geriatric Depression Scale, MoCA = Montreal Cognitive Assessment, MMSE = Mini-Mental State Examination, PD-MCI = Mild cognitive impairment in Parkinson's disease.

a Independent *t*-test.

b Mann-Whitney *U* test.

**Table 2:** Regression coefficients of predictors of cognitive impairment.

	β	SE	p	OR	95% CI for OR	
					Lower Bound	Upper Bound
***Univariate analysis***						
*Age*	0.1	0.0	<**0.001**	1.1	1.1	1.1
*Sex (male)*	0.5	0.3	0.120	1.6	0.9	3.1
*Education*	−1.5	0.3	<**0.001**	0.2	0.1	0.4
*MDS-UPDRS III*	0.1	0.0	<**0.001**	1.1	1.0	1.1
*LEDD (mg/d)*	0.0	0.0	0.941	1.0	1.0	1.0
*GDS-15*	0.5	0.4	0.208	1.6	0.8	3.3
*Anxiety*	0.8	0.4	**0.035**	2.3	1.1	5.0
***Full model***						
*Age*	0.1	0.0	<**0.001**	1.1	1.1	1.1
*Education*	−1.0	0.4	**0.007**	2.7	1.3	5.5
*MDS-UPDRS III*	0.1	0.0	**0.001**	1.1	1.0	1.1
*Anxiety*	1.1	0.5	**0.015**	3.0	1.2	7.3

Significant results highlighted in bold.

Cognitive impairment in Parkinson's disease using the 1 SD cut-off, MDS-UPDRS III = Movement Disorders Society-Unified Parkinson's Disease Rating Scale Part III, LEDD = Levodopa equivalent daily dose, GDS-15 = Geriatric Depression Scale, SE = Standard error, OR = Odds ratio, CI = Confidence interval.

**Table 3:** Regression coefficients of predictors of impaired cognitive domains.

	β	SE	p	OR	95% CI for OR	
					Lower Bound	Upper Bound
***Attention*** (***n = 78)***						
*Age*	0.1	0.0	**0.003**	1.1	1.0	1.1
*Education (years)*	−0.9	0.3	**0.007**	0.4	0.2	0.8
*MDS-UPDRS III*	0.0	0.0	0.074	1.0	1.0	1.1
*Anxiety*	0.3	0.4	0.433	1.4	0.6	2.9
***Memory (n = 89)***						
*Age*	0.1	0.0	**0.005**	1.1	1.0	1.1
*Education (years)*	−0.5	0.3	0.108	0.6	0.3	1.1
*MDS-UPDRS III*	0.1	0.0	**0.001**	1.1	1.0	1.1
*Anxiety*	0.8	0.4	**0.041**	2.3	1.0	5.1
***Executive function (n = 94)***						
*Age*	0.1	0.0	**0.001**	1.1	1.0	1.1
*Education (years)*	−1.2	0.3	**0.001**	0.3	0.2	0.6
*MDS-UPDRS III*	0.0	0.0	**0.029**	1.0	1.0	1.1
*Anxiety*	0.2	0.4	0.713	1.2	0.5	2.6
***Visuospatial function (n = 24)***						
*Age*	0.1	0.0	**0.007**	1.1	1.0	1.1
*MDS-UPDRS III*	0.1	0.0	**0.001**	1.1	1.0	1.1
*Anxiety*	−0.1	0.6	0.888	0.9	0.3	2.9
***Language (n = 55)***						
*Education (years)*	−1.3	0.3	<**0.001**	0.3	0.1	0.5
*Anxiety*	0.2	0.4	0.545	1.3	0.6	2.6

Initial covariates included in each model: age, sex, education, MDS-UPDRS III, LEDD, GDS-15.

Significant results highlighted in bold.

Cognitive impairment in Parkinson's disease using the 1 SD cut-off, MDS-UPDRS III = Movement Disorders Society-Unified Parkinson's Disease Rating Scale Part III, LEDD = Levodopa equivalent daily dose, GDS-15 = Geriatric Depression Scale, SE = Standard error, OR = Odds ratio, CI = Confidence interval.
