# Supplementary material for: Anxiety is associated with cognitive impairment in newly-diagnosed Parkinson's disease
Source: Parkinsonism Relat Disord. 2017 Mar;36:63–8. doi: 10.1016/j.parkreldis.2017.01.001 (PMC5338650; doi:10.1016/j.parkreldis.2017.01.001)
Supplement: Supplementary Table 1 [file mmc1.docx]

**Supplementary Table 1: Comparison of neuropsychological test scores**

|  | **Not prescribed anxiolytics and/or antidepressants (n=32)** | | **Prescribed anxiolytics and/or antidepressants (n=15)** | | **Z** | **p-value** |
| --- | --- | --- | --- | --- | --- | --- |
|  | **Mean** | **SD** | **Mean** | **SD** |  |  |
| *MoCA* | 25.8 | 2.9 | 25.8 | 1.7 | -0.6 | 0.563 |
| *MMSE* | 28.7 | 1.4 | 28.9 | 1.2 | -0.3 | 0.794 |
| *Phonemic fluency* | 10.5 | 4.0 | 13.0 | 4.6 | -1.7 | 0.092 |
| *Semantic fluency* | 22.0 | 7.1 | 23.5 | 6.1 | -0.9 | 0.360 |
| *PoA* | 1389.7 | 295.7 | 1442.1 | 203.8 | -1.6 | 0.108 |
| *Digit vigilance* | 92.3 | 12.5 | 96.1 | 3.3 | -0.4 | 0.681 |
| *PRM* | 19.2 | 4.0 | 17.9 | 2.8 | -1.6 | 0.103 |
| *SRM* | 15.4 | 2.1 | 15.1 | 2.1 | -0.5 | 0.631 |
| *PAL* | 1.8 | 0.5 | 2.1 | 0.6 | -1.5 | 0.143 |
| *OTS* | 15.4 | 2.7 | 14.5 | 3.4 | -0.6 | 0.546 |

SD = Standard deviation; MoCA = Montreal Cognitive Assessment, MMSE = Mini Mental State Examination, PoA = Power of attention, PRM = Paired Recognition Memory, SRM = Spatial Recognition Memory, PAL = Paired Associated Learning, OTS = One Touch Stockings.
