# Supplementary material for: Anxiety is associated with cognitive impairment in newly-diagnosed Parkinson's disease
Source: Parkinsonism Relat Disord. 2017 Mar;36:63–8. doi: 10.1016/j.parkreldis.2017.01.001 (PMC5338650; doi:10.1016/j.parkreldis.2017.01.001)
Supplement: Supplementary Table 2 [file mmc2.docx]

Supplementary Table 2: Regression coefficients of predictors of PD-MCI

|  |  | Β | SE | p | OR | 95% CI for OR | |
| --- | --- | --- | --- | --- | --- | --- | --- |
|  |  |  |  |  |  | Lower  Bound | Upper Bound |
| ***PD-MCI 1.5 SD*** | |  |  |  |  |  |  |
| ***Univariate analysis*** | |  |  |  |  |  |  |
|  | *Age* | 0.05 | 0.02 | **0.003** | 1.05 | 1.02 | 1.09 |
|  | *Sex (female)* | 0.19 | 0.31 | 0.530 | 1.21 | 0.66 | 2.23 |
|  | *Education* | -1.75 | 0.33 | **<0.001** | 0.17 | 0.09 | 0.33 |
|  | *MDS-UPDRS III* | 0.05 | 0.01 | **<0.001** | 1.05 | 1.03 | 1.08 |
|  | *LEDD (mg/d)* | 0.00 | 0.00 | 0.181 | 1.00 | 1.00 | 1.00 |
|  | *GDS-15* | 0.48 | 0.36 | 0.175 | 1.62 | 0.81 | 3.27 |
|  | *Anxiety* | -0.01 | 0.34 | 0.981 | 0.99 | 0.51 | 1.94 |
| ***Full model*** | |  |  |  |  |  |  |
|  | *Age* | 0.05 | 0.02 | **0.015** | 1.05 | 1.01 | 1.09 |
|  | *Education* | -1.60 | 0.35 | **<0.001** | 0.20 | 0.10 | 0.40 |
|  | *MDS-UPDRS III* | 0.05 | 0.01 | **0.001** | 1.05 | 1.02 | 1.08 |
|  | *Anxiety* | -0.07 | 0.41 | 0.862 | 0.93 | 0.42 | 2.07 |
| ***PD-MCI 2 SD*** | |  |  |  |  |  |  |
| ***Univariate analysis*** | |  |  |  |  |  |  |
|  | *Age* | 0.03 | 0.02 | 0.112 | 1.03 | 0.99 | 1.07 |
|  | *Sex (female)* | 0.19 | 0.38 | 0.618 | 1.21 | 0.57 | 2.55 |
|  | *Education* | -1.26 | 0.40 | **0.002** | 0.28 | 0.13 | 0.63 |
|  | *MDS-UPDRS III* | 0.03 | 0.01 | **0.025** | 1.03 | 1.00 | 1.06 |
|  | *LEDD (mg/d)* | 0.00 | 0.00 | 0.554 | 1.00 | 1.00 | 1.00 |
|  | *GDS-15* | 0.81 | 0.40 | **0.045** | 2.24 | 1.02 | 4.95 |
|  | *Anxiety* | -0.16 | 0.42 | 0.707 | 0.85 | 0.37 | 1.96 |
| ***Full model*** | |  |  |  |  |  |  |
|  | *Education* | -1.10 | 0.42 | **0.008** | 0.33 | 0.15 | 0.75 |
|  | *MDS-UPDRS III* | 0.02 | 0.02 | 0.280 | 1.02 | 0.99 | 1.05 |
|  | *GDS-15* | 0.74 | 0.44 | 0.096 | 2.09 | 0.88 | 4.97 |
|  | *Anxiety* | -0.44 | 0.46 | 0.349 | 0.65 | 0.26 | 1.61 |

*Significant results highlighted in bold*

*PD-MCI = Mild cognitive impairment in Parkinson’s disease using the 1 SD cut-off, MDS-UPDRS III = Movement Disorders Society-Unified Parkinson’s Disease Rating Scale Part III, LEDD = Levodopa equivalent daily dose, GDS-15 = Geriatric Depression Scale, SE = Standard error, OR = Odds ratio, CI = Confidence interval*
