# Supplementary material for: Anxiety is associated with cognitive impairment in newly-diagnosed Parkinson's disease
Source: Parkinsonism Relat Disord. 2017 Mar;36:63–8. doi: 10.1016/j.parkreldis.2017.01.001 (PMC5338650; doi:10.1016/j.parkreldis.2017.01.001)
Supplement: Supplementary Table 3 [file mmc3.docx]

Supplementary Table 3: Regression coefficients of predictors of PD-MCI cognitive domains

|  |  | **β** | **SE** | **p** | **OR** | **95% CI for OR** | |
| --- | --- | --- | --- | --- | --- | --- | --- |
|  |  |  |  |  |  | **Lower Bound** | **Upper Bound** |
| ***PD-MCI 1.5SD*** | |  |  |  |  |  |  |
| ***Attention (n=59)*** | |  |  |  |  |  |  |
|  | *Age* | 0.05 | 0.02 | **0.044** | 1.05 | 1.00 | 1.10 |
|  | *Education (years)* | -2.00 | 0.53 | **0.000** | 0.14 | 0.05 | 0.39 |
|  | *GDS-15* | 1.16 | 0.49 | **0.018** | 3.20 | 1.22 | 8.43 |
|  | *Anxiety* | -1.04 | 0.60 | 0.081 | 0.35 | 0.11 | 1.14 |
| ***Memory (n=62)*** | |  |  |  |  |  |  |
|  | *MDS-UPDRS III* | 0.05 | 0.02 | **0.004** | 1.05 | 1.02 | 1.09 |
|  | *Anxiety* | -0.44 | 0.52 | 0.392 | 0.64 | 0.23 | 1.77 |
| ***Executive function (n=58)*** | |  |  |  |  |  |  |
|  | *Age* | 0.07 | 0.03 | **0.012** | 1.07 | 1.01 | 1.13 |
|  | *Education (years)* | -2.34 | 0.64 | **0.000** | 0.10 | 0.03 | 0.34 |
|  | *Anxiety* | -0.38 | 0.58 | 0.510 | 0.68 | 0.22 | 2.13 |
| ***PD-MCI 2SD*** | |  |  |  |  |  |  |
| ***Attention (n=43)*** | |  |  |  |  |  |  |
|  | *Education (years)* | -1.20 | 0.69 | 0.082 | 0.30 | 0.08 | 1.16 |
|  | *GDS-15* | 1.59 | 0.62 | **0.010** | 4.91 | 1.46 | 16.50 |
|  | *Anxiety* | -0.57 | 0.73 | 0.435 | 0.57 | 0.13 | 2.37 |

Initial covariates included in each model: age, sex, education, MDS-UPDRS III, LEDD, GDS-15

Significant results highlighted in bold

Model assumptions for predictors of visuospatial function (n=22) and language (n=22) using PD-MCI 1.5 SD cut off, and memory (n=33), executive function (n=36), visuospatial function (n=3) and language (n=11) using PD-MCI 2 SD cut off were violated; models are not shown.

PD-MCI = Mild cognitive impairment in Parkinson’s disease, MDS-UPDRS III = Movement Disorders Society-Unified Parkinson’s Disease Rating Scale Part III, LEDD = Levodopa equivalent daily dose, GDS-15 = Geriatric Depression Scale, SE = Standard error, OR = Odds ratio, CI = Confidence interval
